# Ultra‐Specific Isolation of Circulating Tumor Cells Enables Rare‐Cell RNA Profiling

**DOI:** 10.1002/advs.201600063

**Published:** 2016-04-18

**Authors:** Rhonda M. Jack, Meggie M. G. Grafton, Danika Rodrigues, Maria D. Giraldez, Catherine Griffith, Robert Cieslak, Mina Zeinali, Chandan Kumar Sinha, Ebrahim Azizi, Max Wicha, Muneesh Tewari, Diane M. Simeone, Sunitha Nagrath

**Affiliations:** ^1^ Department of Chemical Engineering3074 H.H. Dow Building2300 Hayward St.Ann ArborMI48109‐2136USA; ^2^ Biointerfaces InstituteNorth Campus Research Complex2800 Plymouth Road, Bldg. 90Ann ArborMI48109‐2800USA; ^3^ Department of Biomedical Engineering1107 Carl A. Gerstacker Building, 2200 Bonisteel, Blvd.Ann ArborMI48109USA; ^4^ Department of Internal MedicineDivision of Hematology/Oncology and Division of Molecular Medicine and GeneticsUniversity of Michigan109 Zina Pitcher Place, 2061 BSRB ‐ SPC 2200Ann ArborMI48109USA; ^5^ Medical FacultyUniversity of HeidelbergTheodor‐Kutzer‐Ufer1‐3 68167MannheimGermany; ^6^ Michigan Center for Translational PathologyDepartment of PathologyUniversity of Michigan 5309 CCC 5940400 E. Medical Center Dr.Ann ArborMI48109‐0940USA; ^7^ Comprehensive Cancer CenterUniversity of Michigan Health Systems1500 E. Medical Center DrAnn ArborMI48109USA; ^8^ Center for Computational Medicine and BioinformaticsUniversity of MichiganRoom 2017, Palmer Commons, 100 Washtenaw AvenueAnn ArborMI48109‐2218USA; ^9^ Department of SurgeryUniversity of Michigan Health Systems1500 E. Medical Center DrAnn ArborMI48109USA

**Keywords:** cell sorting, immunomagnetism, microfluidics, mRNA, tumor cells

## Abstract

**The clinical potential of circulating tumor cells (CTCs)** in managing cancer metastasis is significant. However, low CTC isolation purities from patient blood have hindered sensitive molecular assays of these rare cells. Described herein is the ultra‐pure isolation of CTCs from patient blood samples and how this platform has enabled highly specific molecular (mRNA and miRNA) profiling of patient CTCs.

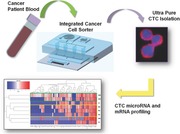

Not surprisingly CTC biology has sparked burgeoning interest within recent years because of the postulated role of these rare cells in metastasis. CTCs initiate from the primary tumor and migrate in the blood and then occupy a secondary site to subsequently metastasize.^1^ They inherently carry information about the tumor that could be useful for understanding metastasis and developing novel therapeutics to more effectively treat these potentially harmful cells. Since CTCs can be isolated from a simple blood draw, they can be routinely used to yield insight into tumor pathology and treatment response without invasive procedures.^2^

However, CTC detection and isolation from whole blood is quite challenging. First, CTCs are rare; there may be only one CTC in 7.5 mL of blood containing billions of blood cells. Second, there is limited knowledge of specific CTC markers, due in part to CTC heterogeneity such that they vary phenotypically and genotypically among patients.^3^ Considering CTC rarity and heterogeneity, engineering a device to isolate them from blood presents an interesting, important challenge.

Two chief approaches used for CTC collection are immunoaffinity capture and label free, size‐based filtering.^4^ Immune‐based methods commonly rely on expression of epithelial cell adhesion molecule (EpCAM), a surface protein, to isolate CTCs and typically require low flow rates (1–3 mL h^−1^) to ensure that CTCs have sufficient time to be immobilized by functionalized surfaces.^5^, ^6^ Issues concerning cell release of the immobilized cells also arise.^7^ In contrast label‐free isolation does not rely on specific biomarkers but instead on inherent CTC properties such as size, deformability, or dielectric susceptibility. Size‐based sorting usually offers higher throughputs since bulk sorting is used.

Moreover, since the discovery of microfluidic inertial size‐based sorting, it was reported that enhanced sorting is achieved by incorporating curvature into microfluidic channels. Curvature introduces Dean forces that augment the inertial forces acting on particles under flow and can generally be performed with high flow rates ranging from 500 to 2000 μL min^−1^, while maintaining typical microfluidic dimensions (see Section S2, Supporting Information).^8^, ^9^, ^10^, ^11^ Whereas label‐free methods are rapid, specificity is lacking and operation is limited by overall high blood cell counts. The recently reported CTC iChip uses hydrodynamic, inertial, and magnetic sorting to isolate CTCs from whole blood.^12^ A throughput of 8 mL h^−1^ using positive immunomagnetic selection, with an average of 1500 contaminating white blood cells (WBCs) from 1 mL whole blood were achieved. Purity was affected by non‐specific WBC labeling with magnetic beads.

We report the development of an ultra‐specific microfluidic device that combines inertial microfluidics and immuno‐magnetism in a uniquely designed workflow that minimizes non‐specific cell labeling for efficient isolation of CTCs from whole blood with ultra‐high CTC purity rates and high throughput. We hypothesized that beginning the CTC isolation process by rapid inertial pre‐sorting would greatly reduce the number of unwanted cells from whole blood so that micrometer‐scale, on‐chip passive mixing and brief on‐chip incubation would facilitate specific, extensive CTC magnetic bead labeling. Then, ultra‐specific sorting of the pre‐enriched magnetized CTCs could be achieved via magnetic sorting. Specifically, the microfluidic design would allow for whole blood to be processed rapidly yet meticulously on a single chip, requiring no blood preparation or long incubation times. This would minimize the extent of non‐specific binding to promote CTC isolation purity. Unlike most other platforms, our approach negates bulk CTC labeling and instead uses on‐chip CTC‐bead labeling which improves the labeling chances of target cell, allowing magnetization and successful isolation from blood samples (see Table S1, Supporting Information).

The functionality of each component is illustrated in **Figure** **1**, showing cell distribution throughout the device. The first step of inertial sorting involves flowing whole blood into one inlet and phosphate buffered saline (PBS) into the other inlet of the spiral module. The use of two differently sized syringes biases the two flows such that whole blood flows at ≈400 μL min^−1^, while buffer flows at 1200 μL min^−1^. By biasing the flow the higher buffer flow rate readily stabilizes flow prior to the blood sample introduction so that no blood sample is wasted initially due to the flow stabilization process. Concurrently, the blood sample is diluted so that the effect of waste cells on cancer cell flow dynamics, as well as on purity and recoveries is effectively reduced.

**Figure 1:**
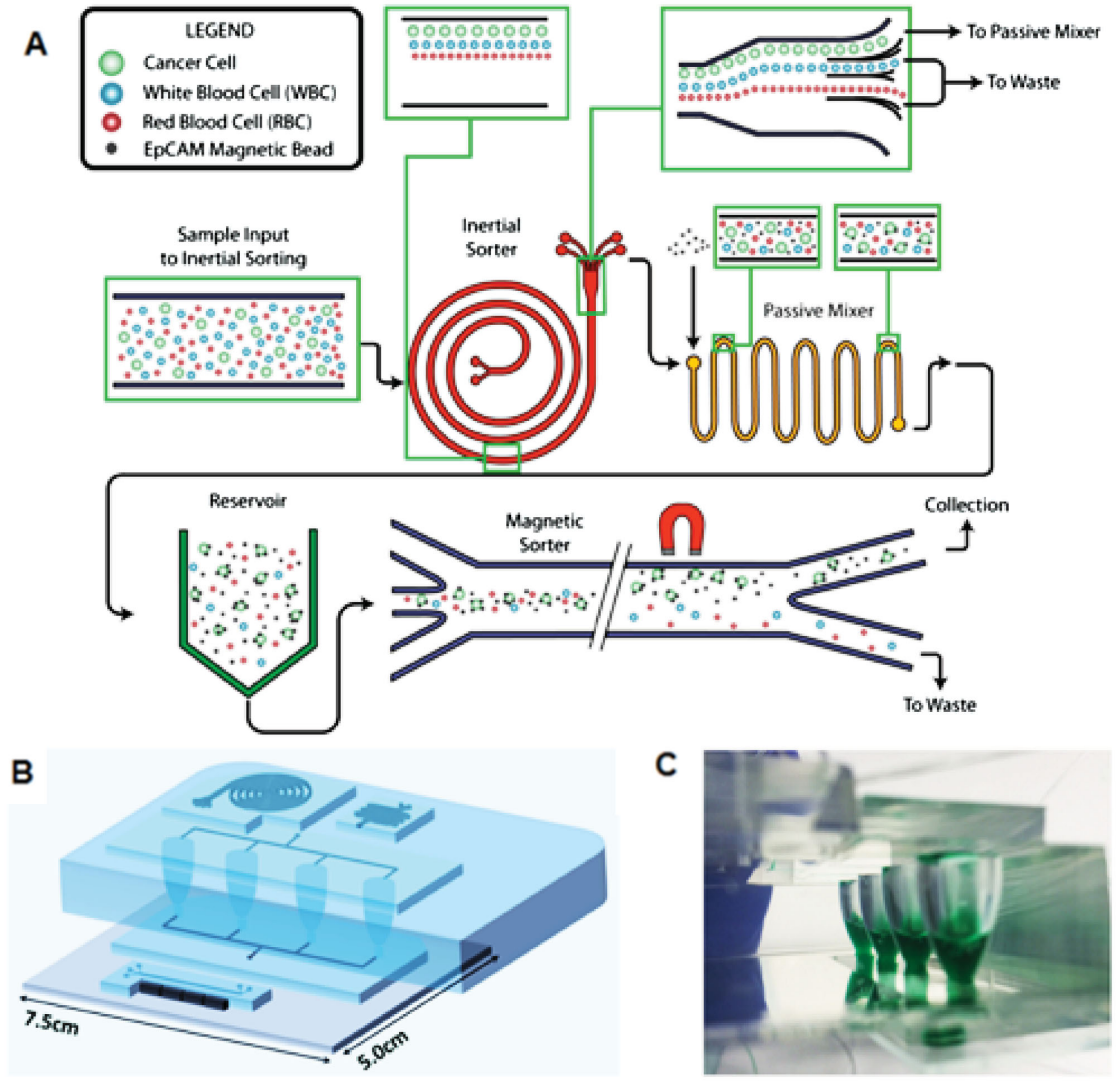
Design of ultra‐specific CTC isolation platform. A) Schematic showing inertial sorting, passive mixing with incubation and magnetic sorting. B) 3D illustration of PDMS device. C) Reservoirs allow for brief incubation and flow rate reduction to enable sensitive magnetic sorting.

Next, the CTC‐enriched stream which leaves the inertial sorter at the innermost outlet, enters the passive mixer where it mixes with EpCAM coated magnetic beads infused at 100 μL min^−1^. After mixing, 5 min on‐chip incubation is carried out in four reservoirs to promote extensive magnetic bead‐CTC labeling via antibody–antigen interactions.

To achieve immediate CTC magnetic sorting after incubation, the reservoir inlet is gently and gradually pressurized with an air‐filled syringe so that the mixture can flow out of the reservoirs in a controlled manner. The mixture drains directly into the magnetic sorter between two buffer streams and permanent magnets lined along the magnetic sorter deflect the magnetically labeled CTCs away from unlabeled blood cells to the CTC collection outlet. The final collection is a highly enriched suspension of viable CTCs.

The inertial sorter separates larger particles (15–20 μm) from smaller particles (7–10 μm). **Figure** **2**A shows the fabricated polydimethylsiloxane (PDMS) device, Figure 2B illustrates the arrangement of cancer cells (green) versus WBCs (blue) at the outlet of the sorter, and Figure 2C demonstrates how the polystyrene particles (20 μm (green), 15 μm (blue), 7 μm (orange)) separate into distinct, focused streams at the outlets of the spiral channel. Similarly in Figure 2D PANC‐1 cancer cells (green streak) focus to the innermost outlet and WBCs (blue streak) focus to the second outlet. Figure 2E shows how the larger PANC‐1 cells (indicated by red arrows) spiked into diluted blood focus to the innermost outlet amidst the other blood cells.

**Figure 2:**
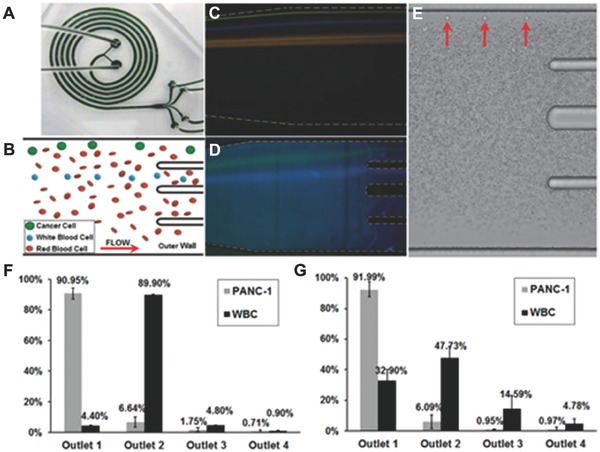
Optimizing inertial sorter module. A) PDMS device. B) Larger CTCs (green) go to innermost outlet channel. C) Fluorescent beads, D) fluorescent cells, and E) high‐speed imaging demonstrate cancer cell sorting. Analysis of cell distribution in outlets both in F) PBS buffer and G) blood.

To quantitatively evaluate inertial sorting PANC‐1 cells and WBCs were spiked into PBS and processed through the module. The effluents from each outlet were collected and analyzed for cell counts. Notably, Figure 2F shows 90.95% ± 3.61 of cancer cells were collected in the desired outlet. Whole blood was routinely diluted by ×10 for blood‐spiked experiments to facilitate conditions amenable to microscope visualizing. Figure 2G shows cell distribution in the four outlets after repeating the experiments in diluted blood. Results consistent with PBS‐spiked experiments were achieved such that 91.99% ± 4.94 of cancer cells were enriched in the innermost spiral outlet. The spiral design was optimized as a stand‐alone device and subsequently, the three waste outlets were re‐designed to allow for resistance‐matching once it was connected within the fully integrated system (see Figure S1 and Table S2, Supporting Information).

The passive mixer facilitates mixing of EpCAM magnetic beads with the CTC‐rich effluent from the spiral sorter so that magnetic beads bind to cancer cells through antibody–antigen complexes. Mixing occurs after inertial whole blood pre‐sorting to increase the chances of bead‐cancer cell interactions, which economizes on the quantity of required antibodies. Additionally, mixing occurs at length‐scales comparable to cancer cell sizes thus promoting efficient bead‐CTC labeling compared to bulk labeling. Because both viscous and inertial forces are appreciable in this range of Re, “secondary flows” arise in both axial and radial directions thus increasing the interfacial area over which diffusion and mixing occur (see Section S3, Supporting Information).^13^ Furthermore, the short mixing time required decreases the likelihood of nonspecific binding with non‐target cells.

Using flow rates of 10–100 μL min^−1^, mixers were first evaluated by testing the distance along various designs required for two distinct streams of yellow‐ and blue‐dyed PBS to become one well‐mixed green stream (see Figure S2, Supporting Information). The highly compact selected mixer design enabled fluids to completely mix by the third channel segment, after ≈4 cm footprint. Next cancer cell labeling with EpCAM beads was examined through static experiments (PANC‐1 cells and EpCAM beads in an Eppendorf tube) and on‐chip experiments (on‐chip passive mixing of cells and beads subsequently flowed into reservoirs). Calculations demonstrate that for a 20 μm cell to experience a sufficient magnetic force to pull it in the direction perpendicular to flow and therefore sort it, less than 1/3 bead coverage is required (see Section S4, Supporting Information). For four incubation time points (5, 10, 15, and 30 min), labeling was evaluated by immediately imaging the mixed effluent to quantify beads attached to cells. Cells were characterized as having no coverage, <1/3, 1/3–2/3, and >2/3 coverage (see Figure S3, Supporting Information). In static conditions 15 min incubation allowed at least 1/3 coverage on only 85% of cells (Figure S6, Supporting Information). Conversely, when cells diluted in blood and beads were mixed on‐chip at 100 μL min^−1^ and allowed 5 min incubation in the reservoirs, >92% of the cells demonstrated over 1/3 coverage. Thus, 5 min of incubation was determined to be optimal for on‐chip labeling. Not only do the reservoirs facilitate on‐chip incubation for extensive cancer cell bead coverage, yet they enable a seamless switch of flow rates from 1200 to 50 μL min^−1^, which in turn facilitates sensitive, thorough magnetic sorting of magnetized cancer cells.

The magnetic sorter has three inlets consisting of two outer buffer streams and one middle sample stream that flow in a 250 μm wide channel and allows for further continuous on‐chip cell sorting.^14^ Confining the sample stream in this manner ultimately provides efficient magnetized CTC sorting from contaminating cells by ensuring that only magnetized cells are attracted into the collection outlet. The sorter outlet is split into two channels, with a smaller collection (35 μm) and a larger waste (65 μm) channel. Permanent magnets (500 mT, K&J Magnetics) aligned end‐to‐end, are positioned parallel to the main channel with a 1 mm PDMS separation gap between the channel and the magnets. The sample continuously flows through the sorter at 50 μL min^−1^ so that cells with as few as 1/3 surface bead coverage are deflected into the collection channel. Waste cells continue uninterrupted to the waste outlet.

In determining the appropriate magnetic sorting flow rates, PANC‐1 cells pre‐labeled with magnetic beads were flowed through the sorter at test rates of 25–100 μL min^−1^, with top (farther from magnet) buffer streams of 15–50 μL min^−1^ and bottom (closer to magnet) buffer streams of 25–75 μL min^−1^. Biasing the flow rates was readily achieved by using two different size syringes at a fixed syringe drive setting so that the top and bottom streams could simultaneously flow at two different flow rates. Notably, biasing the flow in this manner significantly improved magnetic sorting by allowing magnetized cells to flow slower to maximize magnetic trapping and to enhance separation distance of waste cells from the collection channel (see Video S1, Supporting Information). Using 75 μL min^−1^ bottom, 25 μL min^−1^ top buffer, and 50 μL min^−1^ flow rates, results show that for blood spiked tests, WBC contamination was 2.28% ± 0.68 and recovery efficiency was 94.60% ± 1.48. Of note, the few PANC‐1 cells in the waste were poorly labeled and so not expected to be magnetically sorted (Figure S6, Supporting Information).

To establish the proof‐of‐concept of the device, 1 mL whole blood was routinely processed. PANC‐1 cells spiked at 100 and 1000 cells mL^−1^ were used to evaluate device performance with cancer cells. Results demonstrate that efficiency of PANC‐1 collection was nearly 90% (**Figure** **3**C). For both cases, contamination ranged from 82 to 801 WBCs mL^−1^ (Figure 3D), resulting in purities of up to 75%. The 3‐(4,5‐dimethylthiazol‐2‐yl)‐2,5‐diphenyltetrazolium bromide (MTT) colorimetric assay was used to assess isolated PANC‐1 cell viability which revealed that cells processed through the integrated system had viability rates comparable to that of the unprocessed control cells thus supporting the feasibility of culturing the collected cells (Figure S8, Supporting Information). Also, in consideration of developing a point‐of‐care diagnostic device, the antibody‐labeled beads were tested and demonstrated stability in cancer cell labeling even after 100 d (see Section S7, Supporting Information). The unprecedented purity rates achieved by the device supersede some of the other reported technologies including GEDI (68%), herringbone (14%), iChip (1500 WBCs mL^−1^), and the CTC chip (9%)^5^, ^12^, ^15^, ^16^ and facilitates CTC molecular profiling.

**Figure 3:**
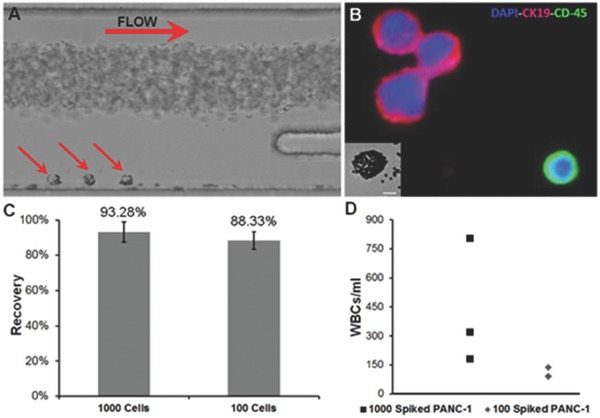
Integrated device operation. A) Magnetic sorter outlets show highly enriched cancer cells separating from waste cells. B) Immunofluorescence staining used in identifying cancer cells (PANC‐1) and WBCs with inset of magnetically labeled cancer cell (10 μm scale bar). C) Recovery of PANC‐1 cells spiked into whole blood at 100 and 1000 cells mL^−1^. D) Number of contaminating WBCs for 100 and 1000 PANC‐1 cells spiked into whole blood.

14 metastatic or locally advanced PDAC patient samples and four healthy controls were processed through the device (**Figure** **4**A). Up to 1.4 mL of whole blood from each patient was processed and CTCs were identified via fluorescent immunostaining for CK+/DAPI+/CD45−. Additional confirmation of an epithelial cell type was garnered by brightfield images showing bound EpCAM magnetic beads on the cells. WBCs were identified as CK−/DAPI+/CD45+. Figures 4D and 5E are representative images of fluorescently labeled CTCs and WBCs, respectively. Samples were enumerated and CTC purity percentages are as reported in **Figure** **5**C. The corresponding patient clinical data are shown in Table S3 (Supporting Information). For all 14 patients, CTCs were isolated with counts ranging from 14 to 938 CTCs mL^−1^ which was higher than the average 3 CTCs mL^−1^ for healthy controls. Notably, CTCs have been recovered from patient blood with significant depletion, on the order of 10^6^ WBCs with average CTC purity being 82.5% ± 23.5.

**Figure 4:**
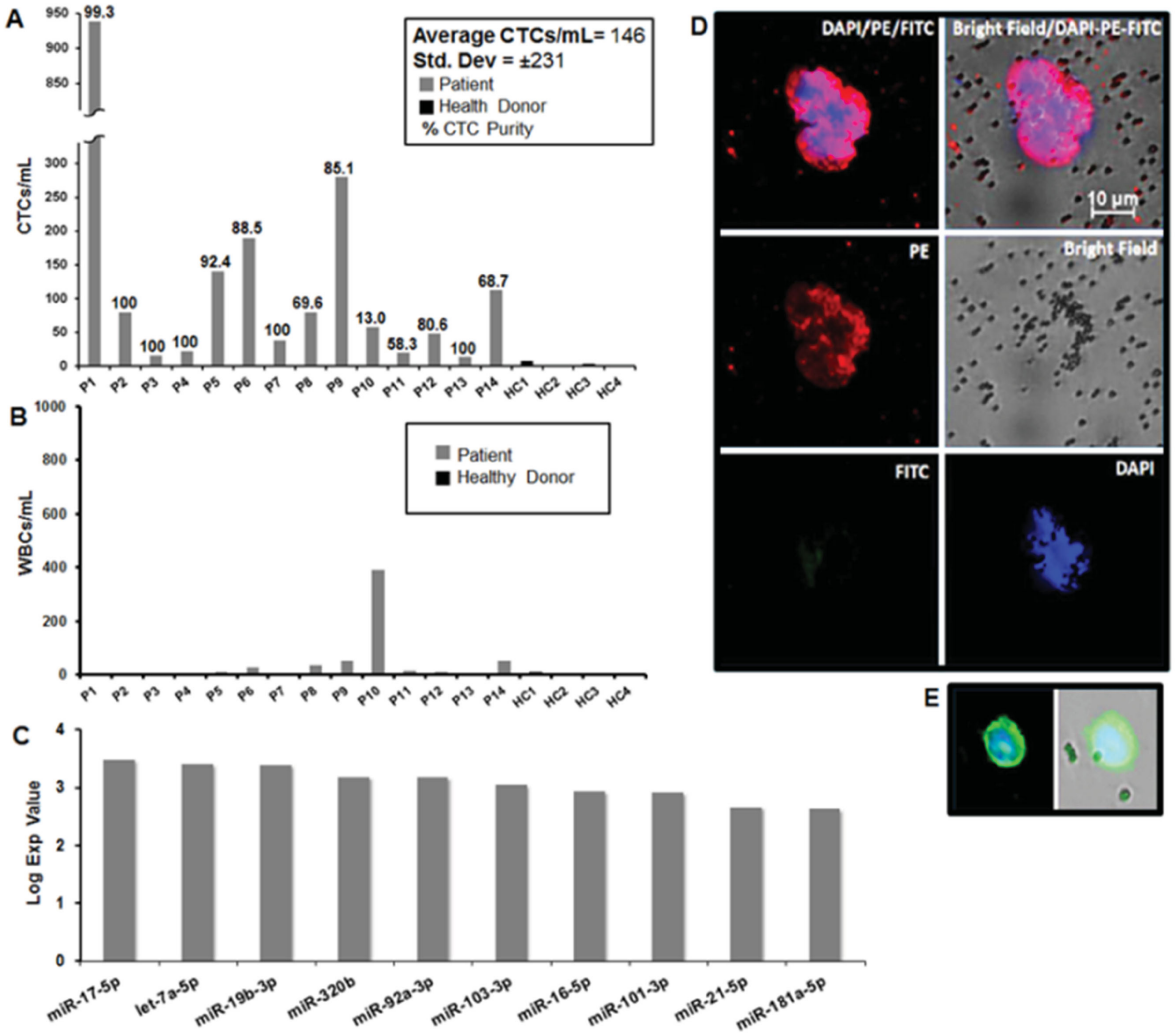
Processing PDAC samples. A) Cell counts of CTCs in 1 mL of patient blood. Percent purities are indicated. B) Corresponding WBCs for the same samples. C) Log exp values for ten highest expressed miRNAs. D) Fluorescent and brightfield image of CTCs and E) WBCs identified through immunofluorescent staining (10 μm scale bar).

**Figure 5:**
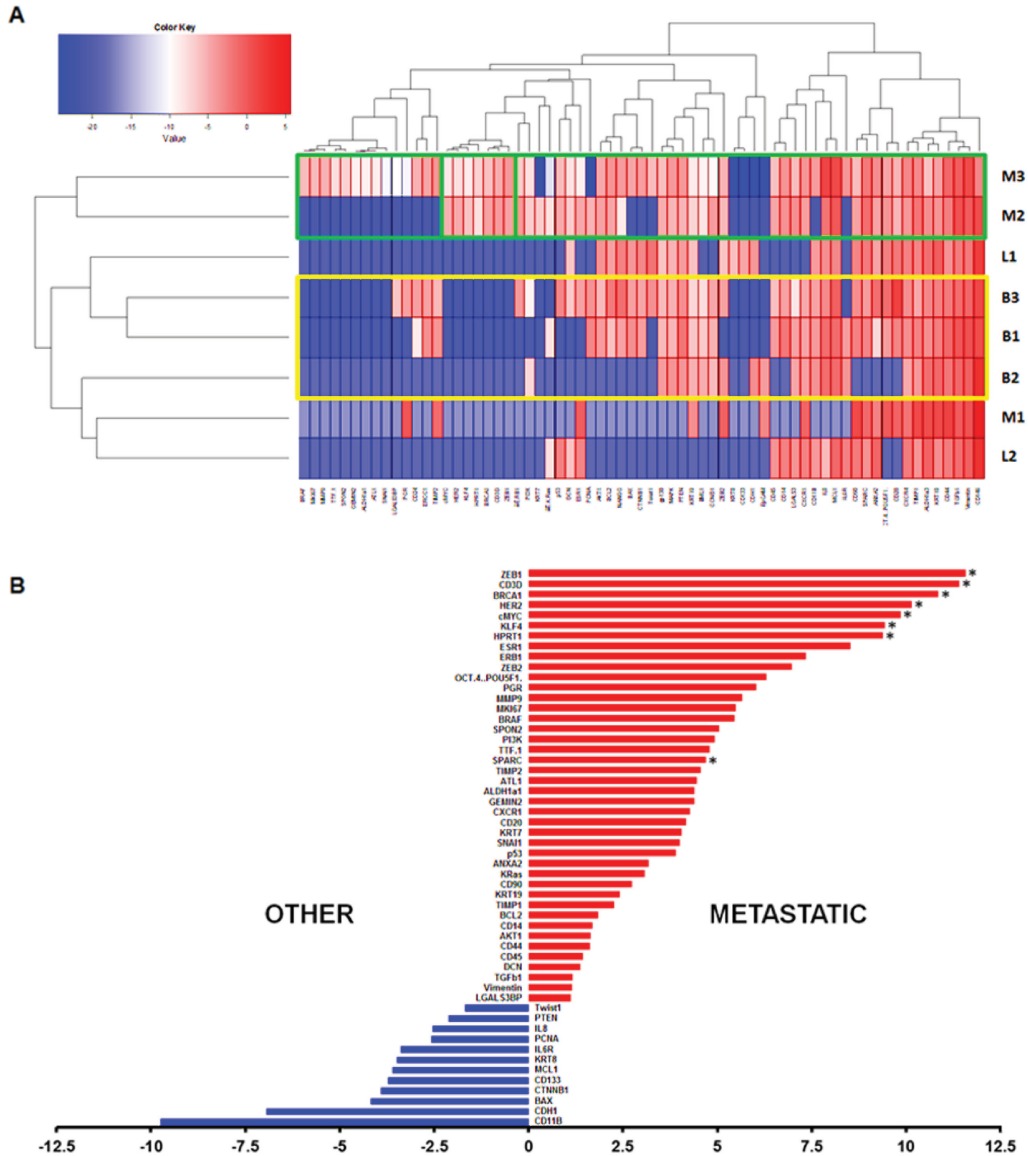
Messenger RNA profiling of CTC enriched PDAC samples from three borderline (B) and two locally advanced (L) patients as well as three metastatic patients (M). A) Heat map plot reveal that two metastatic profiles cluster together as a separate group from others excluding one outlier patient. Seven genes (green box) distinguish between the two groups. B) Log fold increase plot shows mRNAs increased in borderline versus metastatic patients. (* indicates samples with *p* = 0.036.)

Although magnetic sorting is the rate limiting process, occurring at 50 μL min^−1^, it can easily be multiplexed. Using parallel sorters (*n* = 8) on a single chip, the entire process can be maintained at 400 μL min^−1^, yielding an overall processing rate of greater than 20 mL h^−1^ and still yield unprecedented purities of greater than 80%.

MiRNAs are important regulators of cancer cell properties; however, they have been, in general, understudied in the context of CTCs. Currently, the relatively low purity rates proffered by most CTC isolation technologies limit specificity in genomic characterizing. The high CTC purities of our device enable CTC‐specific miRNA profiling. Accordingly we profiled 372 miRNAs in CTC enriched samples from two locally advanced PDAC patients alongside a healthy control individual.

Of the 372 miRNAs profiled, 106 miRs demonstrated abundance in expression in both samples compared to the healthy control. 81 miRs had low expression and were categorized as not reliably detected. Interestingly, several miRs previously reported as associated with PDAC including, miR‐221‐3p, 210, ‐23a‐3p, ‐143‐3p, and ‐21‐5p were among those highly expressed in the CTC samples (Figure 4C).^17^, ^18^, ^19^, ^20^ Additionally, based on pathway analysis, the top network functions enriched in the profiles were for cancer and organismal injury and abnormalities. Pathways involving Smad2/3 and MAP2K1/2 were implicated in PDAC CTC biology (see Figure S9, Supporting Information). Moreover, for the first time we report that PDAC CTC miRNA profiling revealed a trend toward high expression of miRs 17‐5p, 19b‐3p, 320b, and let7a‐5p. Also, miR‐96‐3p, 216b, 155‐5p, 212‐3p, and ‐31‐5p which demonstrated decreased expression have been previously reported to exhibit down regulation in pancreatic cancer.^17^, ^18^

To further demonstrate the potential of the integrated device in allowing sensitive molecular interrogation of CTC samples subsequent to CTC isolation, mRNA profiling was carried out on eight additional patient samples. Of these, three were categorized as borderline and two as locally advanced while three were metastatic. When metastatic profiles were grouped against other patients, as seen in Figure 5A, all borderline profiles clustered closely while two of three metastatic cases clustered apart from all other profiles. Moreover, seven mRNAs Zeb1, KLF4, cMYC, BRCA1, HER2, CD3D, and HPRT1 levels (indicated by the green box in Figure 5A) were able to distinguish among the two groups. SPARC was also higher‐expressed in the metastatic group (LogFC = 4.7, *p* = 0.037). Specifically, Zeb1 (LogFC = 11.6, *p* = 0.037) has shown to be an independent predictor of mortality in pancreatic cancer while the cause of overexpression of cMYC (LogFC = 9.9, *p* = 0.037) in pancreatic cancers has been previously studied.^21^, ^22^ The role of KLF4 (LogFC = 11.6, *p* = 0.037) as both as a tumor suppressor and an oncogene in cancer progression was previously reported.^23^ Interestingly, BRCA1 (LogFC = 10.9, *p* = 0.037) was reported to be down regulated in cases of pancreatitis and sporadic PDAC.^24^ Additionally, the results of HER2 amplification (LogFC = 10.1, *p* = 0.037) among PDAC patients showed potential for therapeutic endeavors.^25^ Aberrantly high expression of SPARC (LogFC = 4.7, *p* = 0.037) in pancreatic CTCs was also observed in both mouse and human samples.^26^ Taken together, the corroboration of reported studies by the results of CTC molecular profiling enriched by the described device show promise in its use to ascertain information that may be specific to CTCs.

In summary, the integrated microfluidic device described is capable of generating significantly enriched, viable CTCs which enable CTC miRNA and mRNA analysis. The device uses inertial sorting to perform rapid pre‐sorting of the original sample which significantly reduces the amount of sample, thus reducing the overall time required for processing. On‐chip magnetic labeling allows for very sensitive CTC‐bead tagging in the absence of the majority of blood cells and reduces non‐specific binding. By multiplexing the magnetic sorter module, the device can process up to 10 mL of blood in 1 h and is a closed device requiring limited user involvement. Ultra‐pure CTC‐specific RNA profiling revealed abundance in expression of miRs 17‐5p, 19b‐3p, 320b, and let7a‐5p along with mRNAs Zeb1, KLF4, cMYC, BRCA1, HER2, CD3D, and HPRT1 in PDAC derived CTCs. The platform presented here should enable more studies of CTC RNA expression which can in turn, help to identify their putative roles in cancer metastasis.

## Supporting information

As a service to our authors and readers, this journal provides supporting information supplied by the authors. Such materials are peer reviewed and may be re‐organized for online delivery, but are not copy‐edited or typeset. Technical support issues arising from supporting information (other than missing files) should be addressed to the authors.

Supplementary
