# Supplementary material for: Ultra‐Specific Isolation of Circulating Tumor Cells Enables Rare‐Cell RNA Profiling
Source: Adv Sci (Weinh). 2016 Apr 18;3(9):1600063. doi: 10.1002/advs.201600063 (PMC5039969; doi:10.1002/advs.201600063)
Supplement: Supplementary file 1 — Supplementary [file ADVS-3-0e-s001.pdf]

Copyright WILEY-VCH Verlag GmbH & Co. KGaA, 69469 Weinheim, Germany, 2016.

ADVANCED SCIENCE
Open Access

Supporting Information

for *Adv. Sci.,* DOI: 10.1002/advs.201600063

Ultra-Specific Isolation of Circulating Tumor Cells Enables Rare-Cell RNA Profiling

*Rhonda M. Jack, Meggie M. G. Grafton, Danika Rodrigues, Maria D. Giraldez, Catherine Griffith, Robert Cieslak, Mina Zeinali, Chandan Kumar Sinha, Ebrahim Azizi, Max Wicha, Muneesh Tewari, Diane M. Simeone, and Sunitha Nagrath**

Copyright WILEY-VCH Verlag GmbH & Co. KGaA, 69469 Weinheim, Germany, 2013.

Supporting Information

Ultra-Specific Isolation of Circulating Tumor Cells Enables Rare-Cell RNA Profiling

Rhonda M. Jack, Meggie M. G. Grafton, Danika Rodrigues, Maria D. Giraldez, Catherine Griffith, Robert Cieslak, Mina Zenali, Chandan Kumar-Sinha, Ebrahim Azizi, Max Wicha, Muneesh Tewari, Diane M. Simeone, and Sunitha Nagrath*

Contents

Section 1. Methods.

Section 2. Theory of Inertial Sorting.

Section 3. Passive Mixing in Serpentine Channel.

Section 4. Magnetic Bead Coverage Efficiency.

Section 5. Experimental Labeling Efficiency and magnetic cell sorting.

Section 6. Bead/Antibody Reactivity.

Section 7. Cell viability.

Figure S1. Inertial Sorter Optimization

Figure S2. Mixing progression through passive mixer.

Figure S3. *Comsol* calculation of magnetic field strength at channel edges.

Figure S4. Minimum bead coverage required for magnetic collection.

Figure S5. Magnetic bead labeling efficiency of PANC-1 cells.

Figure S6. Incubation time required for bead-cell labeling.

Figure S7. Magnetic sorter operation.

Figure S8. Examination of the prolonged reactivity of antibody coated beads.

Figure S9. MTT assay to determine cell viability.

Figure S10. Network generated in IPA from CTC MiRNA profiling.

Table S1. Comparison of technologies with the reported device for CTC sorting and isolation

Table S2. Parameters and devices studied during the 2nd generation devices to arrive at final spiral design

Table S3. PDAC patient information and status

Table S4. Data for individual patient samples either miRNA or mRNA profiled

Video S1. Magnetic bead-labeled cancer cells being magnetically sorted from the waste stream into the collection outlet to achieve ultra-specific CTC sorting

1. *Experimental Section*

Fabrication of PDMS Microfluidic Chip. Microfluidic channels were designed using AutoCAD and master molds were fabricated using SU8-2100 negative photoresist (Microchem Corp.) following standard photolithography procedures. All molds were fabricated with a thickness of 100µm. A 10:1 ratio of PDMS polymer to curing agent (Dow-Corning) was mixed and de-bubbled prior to pouring over the SU8 molds. The PDMS was allowed to cure at 65^0C overnight. After curing, the PDMS chips were manually cut and peeled from the mold, trimmed to size, and inlet/outlet holes were punched. PDMS chips were bonded by oxygen plasma to cleaned glass slides. Tygon tubing was inserted to connect device inlets and outlet ports to syringes. All flow was driven by Harvard Apparatus syringe pumps. Prior to running samples, tubing and chips were primed with PBS and 1% Pluronic F127 solution for several minutes to prevent cells from sticking to PDMS surfaces.

Polymer Micro Beads. Device prototypes for the spiral inertial sorter and the passive mixer were first evaluated using fluorescent polymer beads. 20µm (FITC, Polymer Sciences), 15µm (DAPI, Invitrogen), 10µm (Dragon Green, Bangs Lab), and 7µm beads (Sky Blue, Spherotech) were used for flow characterization at concentrations ranging from 10^4-10^5 beads mL^{-1}. Also, magnetic fluorescent polymer beads (8µm, Bangs Lab) were used to characterize the magnetic sorter.

Cancer Cells. A PANC-1 cell line (human pancreatic carcinoma, epithelial-like cell line), obtained from ATCC, was maintained at 37°C with 5% $C0^2$ and 95% relative humidity in DMEM, supplemented with 10% FBS. PANC-1 cells were harvested on the third day after seeding, once they achieved >60% confluence, to exploit optimal cell surface EpCAM expression. PANC-1 cells were labeled with CellTracker Green dye (Thermo Fisher Scientific)

for easy visualization and counting. Human white blood cells (WBCs) which were used for spiking experiments were obtained from healthy donor blood using a dextran-mediated, centrifugation technique for WBC isolation. The cells were then fixed and made permeable with BD CytoPerm/CytoFix to enable nuclear staining with DAPI. All fixed cells were stored at 4°C. For appropriate dilutions, cells were counted using a hemocytometer and then diluted with PBS accordingly. For cell spiking experiments of 1000 cells mL^{-1} or less, 1 mL of cells was prepared at 10^4 cells mL^{-1} concentration and 10-100 µL of the solution was added to a 1 mL blood aliquot for device testing. Two additional samples were plated during recovery experiments to be used as counting controls, to supplement mass balance results.

Human Blood Sample Collection and Processing. Blood samples from pancreatic cancer patients were collected using institutional IRB guidelines with informed consent from the patients or the next of kin while blood from healthy donors were collected with informed consent according to a standard protocol. The patient samples, (courtesy of the University of Michigan Comprehensive Cancer Center) and the healthy donor samples were collected in EDTA tubes and tested within six hours of blood draw. For each sample, a maximum of 1.4 mL of whole blood was processed through the integrated microfluidic device to enumerate for CTCs. The sample outputs were collected in microcentrifuge tubes for subsequent immunostaining. For miRNA analysis, 6.5mLs of blood was processed from each patient.

Immunofluorescence Staining of Isolated CTCs. CTCs isolated by the integrated device were processed by cytospinning 250 µL of sample and then fixing with 250 µL of 4% PFA for 10 min. Slides were then stained for CTC markers using a primary antibody for anti-cytokeratin-19 (CK-19, epithelial cell marker, (Santa Cruz Biotechnology, rabbit polyclonal IgG) and nuclear stain 4',6-Diamidino-2-Phenylindole, Dihydrochloride (DAPI, Invitrogen). Negative staining with the

leukocyte marker anti-CD45 (BD Pharmigen Purified Mouse Anti-Human CD45, HI30 clone, isotype Mouse IgG1, ƙ) was also performed. Samples were then stained using secondary antibodies with fluorescence conjugation namely, AlexaFluor 568 (Life Technologies), for CK-19 detection and AlexaFluor 488 (Life Technologies), for CD45 detection. For the primary antibody stains, an overnight incubation period was carried out, while for secondary staining, 1hr was allowed. For DAPI staining ProLong Gold anitfade mountant with DAPI, (Life Technologies) was applied overnight. Immunofluorescence imaging for anti-CD45 was performed using a FITC filter while anti-CK was performed with a PE filter and DAPI, a UV filter.

Patient CTC MicroRNA Profiling. After enriching for CTCs from 6.5 mL of patient blood, 300μL of the fraction was fixed and stained to enumerate for CTC levels and the remaining portion was processed for total RNA isolation. RNA was isolated using the Norgen Single Cell RNA Purification kit (Norgen Biotek Corp.), according to the manufacturer's protocol with minor modifications. After lysing the cells a mix of 3 synthetic Caenorhabditis elegans, (C. elegans) miRNA oligonucleotides, cel-miR-39, cel-miR-54 and cel-miR-238 was added to the sample to be used for miRNA normalization. The CTC lysates were then stored in -80^{0}C until total RNA purification was ready to be carried out. For total RNA purification, samples were thawed on ice and 200μL of 96-100% ethanol was added to the cell lysate and vortex mixed for 10 seconds. At this point the manufacturer's protocol was followed. Once total RNA was extracted, samples were profiled for the abundance of 372 miRNAs. To carry out reverse transcription, 13μL of each sample was processed using miRCURY LNA Universal RT microRNA PCR kit (Exiqon) according to the manufacturer's protocol. UniSP6 spike-in control was added to each reaction for normalization. Once reverse transcription was completed, the

products were diluted and combined with SYBR Green master mix (Exiqon) and ROX reference dye (Applied Biosystems) and loaded into the commercially available, Ready-to Use- PCR, Human panel I, V4.M qRT-PCR arrays (Exiqon). QuantStudio ViiA7 PCR system (Applied Biosystems) was used for carrying out quantitative PCR. CT values were calculated by exporting the data using automatic threshold values. Normalization was carried out across all plates based on the data obtained for the UniSP6 and Inter-Plate Calibrator control assays. The blanks of all the assays were undetectable. Any miRNA assay having a CT value beyond the limit of detection or greater than the blank control array were considered as not detected (ND). All other assays that were reliably detected were median normalized. Based on these CT values, fold change for each miRNA was calculated based on the expression levels in the healthy control. Fold change values were then uploaded to Ingenuity Pathway Analysis (IPA) software (Qiagen), and analysis was carried out using Ingenuity Knowledge base reference set, with filters for Human species alongside an "experimentally observed" confidence.

Patient CTC mRNA Profiling. Total RNA was purified as for miRNA profiling. RT was carried out on the purified RNA followed by pre-amplification (18 cycles) using cDNAs corresponding to 96 genes of interest from a CTC-specific panel. The pooled TaqMan Gene Expression Assays and Cell-to-CT Kit (Ambion, Invitrogen) was used. TaqMan Gene Expression Assays for the 96 genes and the BioMark HD qPCR platform (35 cycles) was used to carry out gene expression profiling of the pre-amplified cDNAs. For genes undetected during qPCR, a cycle number of 40 was assigned for analysis purposes. To normalize Ct values among different genes, GAPDH was used as an internal reference. Statistical analysis was performed based on $-\Delta Ct$ of gene expression.

2. *Inertial Sorting*

Theory of Inertial Sorting[11]. The separation principle of the spiral is based on the concept that particles flowing along a rectangular, curved channel will experience a combination of inertial lift forces, F_L and Dean drag forces, F_D, the magnitudes and directions of which, are dependent on particle size and the relative position of the particle across the channel cross section. These two lift forces are oppositely directed and they result in a net lift force, F_L that focuses different streams of particles within the microchannel. This force is given by

$$F_L=\rho G^2 C_L a_P^4 \quad \text{(S1)}$$

where ρ is the fluid density, G is the fluid shear rate given by $G=U_{max}/D_h$, where U_{max} is the maximum fluid velocity and D_h is the hydraulic radius of the channel. C_L represents the lift coefficient which is a function both of the channel's Reynolds number, *Re* and the particle position along the channel cross section.[10-11] Particles experience a transverse drag force from the Dean vortices, which is calculated by assuming Stokes drag such that

$$F_D=3\pi\mu U_{Dean} a_p \quad (S2)$$

where U_{Dean} is the average Dean velocity given by $U_{Dean}=1.8 \text{ x } 10^{-4} De^{1.63}$ where *De* is the Dean Number given by $De=Re(D_h/2R)^{1/2}$. *R* is the radius of curvature of the channel and μ is the fluid viscosity. For the spiral device studied, the estimated ratio of F_D/F_L for a CTC (a_p ~20μm) is 2.148 while F_D/F_L for a WBC (a_p ~10μm) is 0.468 so that the 2 particles equilibrate at different cross sectional positions. Essentially, the magnitude of the ratio of F_D/F_L which varies with a_p^3 (a_p being particle diameter), determines the equilibrium position adopted by particles of different sizes.[11] Thereupon, the spiral device can be engineered so that particles of different diameters occupy single, focused streams along the channel length. Ideally, in the present study, when a patient's blood sample is flowed through the inertial spiral separator, all of the small blood cells

will be focused to the outer wall thus resulting in a suspension consisting mainly of cancer cells and some WBCs in the innermost channel outlet.

Device Design. The optimized spiral design for the integrated chip consists of a 500 µm wide channel that expands to 1000 µm at the outlet and branches into one collection outlet (innermost outlet, 200 µm wide) and three waste outlets. The largest radius on the spiral is 15 mm and the height of the microfluidic channels is 100 µm. The spiral device operates at 1700 µL min^{-1} as a stand-alone platform, for effective sorting of large (15-20 µm) particles from smaller particles (7-10 µm). Once the inertial sorter is fitted into the integrated device, the spiral operating flow rate is dropped to 1200 µl/min for resistance-matching and to maintain effective CTC sorting.

Table S1: Comparison of technologies with the reported device for CTC sorting and isolation

Table S2 . Comparison of CTC Isolation Platforms

Platform	Inertial Separation	Magnetic Sorting	Microfluidic Magnetic Labeling	Through-put	Cell Line Recoveries	Purities of CTCs	Live Cells	Whole Blood	CTCs/mL Detected	References
Cell Search	No	Yes	No, bulk labeling	N/A	>80	---	No	No	0.16 (Panc)	(Ozkumur, Shah et al. 2013)
CTC iChip	Yes	Yes	No, bulk labeling	9.6mL/hr	98%	7.8%	Yes	Yes	0.59 (Panc)	(Ozkumur, Shah et al. 2013)
Clear Cell	Yes	No	N/A	3mL/hr	>85%	10%	Yes	Yes, dilution required	39.1 (Met Lung)	(Hou, Warkiani et al. 2013)
IsoFlux	No	Yes	No, bulk labeling	1.2mL/hr	85%	2.3%	Yes	No	10.6 (Prostate)	(Harb, Fan et al. 2013)
LiquidBiopsy	No	Yes	No, bulk labeling	5mL/hr	78%	10%	No	Yes	---	(Winer-Jones, Vahidi et al. 2014)
Mag Sweeper	No	Yes	No, bulk labeling	9mL/hr	62%	51%	Yes	Yes, dilution required	1.3 (MBC)	(Talasaz, Powell et al. 2009)
**Presented Device**	**Yes**	**Yes**	**Yes**	**24mL/hr**	**88%**	**83%**	**Yes**	**Yes**	**146 Panc**	---

Table S2. Parameters and devices studied during the 2nd generation of devices to arrive at a final spiral design.

Device #	Spiral Radius (mm)	Outlet Widths (µm)				# of Turns	Comments
		1	2	3	4		
1	15	125	125	125	225	6	500µm wide (75,75,250 spacing)
2	15	150	150	150	350	6	Outlets designed by measurement 500µm wide (50, 50, 50 spacing)
3	20	150	150	250	250	3	500µm wide (50, 100, 50 spacing)
4	10	150	150	250	250	5	100µm wide channel (50, 100, 50 spacing)
5	20	150	150	250	250	3	250µm wide Short expansion (50, 100, 50 spacing)
6	20	150	150	250	---	3	250µm wide, 3 outlets, 700 vs 1000µm expansion (50, 100 spacing)
7	20	150	150	250	250	3	250µm wide channel (50, 100, 50 spacing)
8	20	125	125	125	125	3	250µm wide, 575 vs 1000µm expansion (25, 25, 25 spacing)
9	10	150	150	250	250	5	250µm wide channel (50, 100, 50 spacing)
10	10	150	150	250	250	5	500µm wide channel (50, 100, 50 spacing)
11	15	100(x3)	100	100	100(x3)	6	25µm spacing 8 outlets, 3 grouped 5 single
12	15	150	150	250	250	6	50,100,50 spacing
13	15	150	150	150	350	6	50,100,50 spacing
14	15	200	100	125	150(x2)	6	5 outlets, varied spacing
15	15	100	100	100	100(x3)	6	3 single , 3 wastes grouped

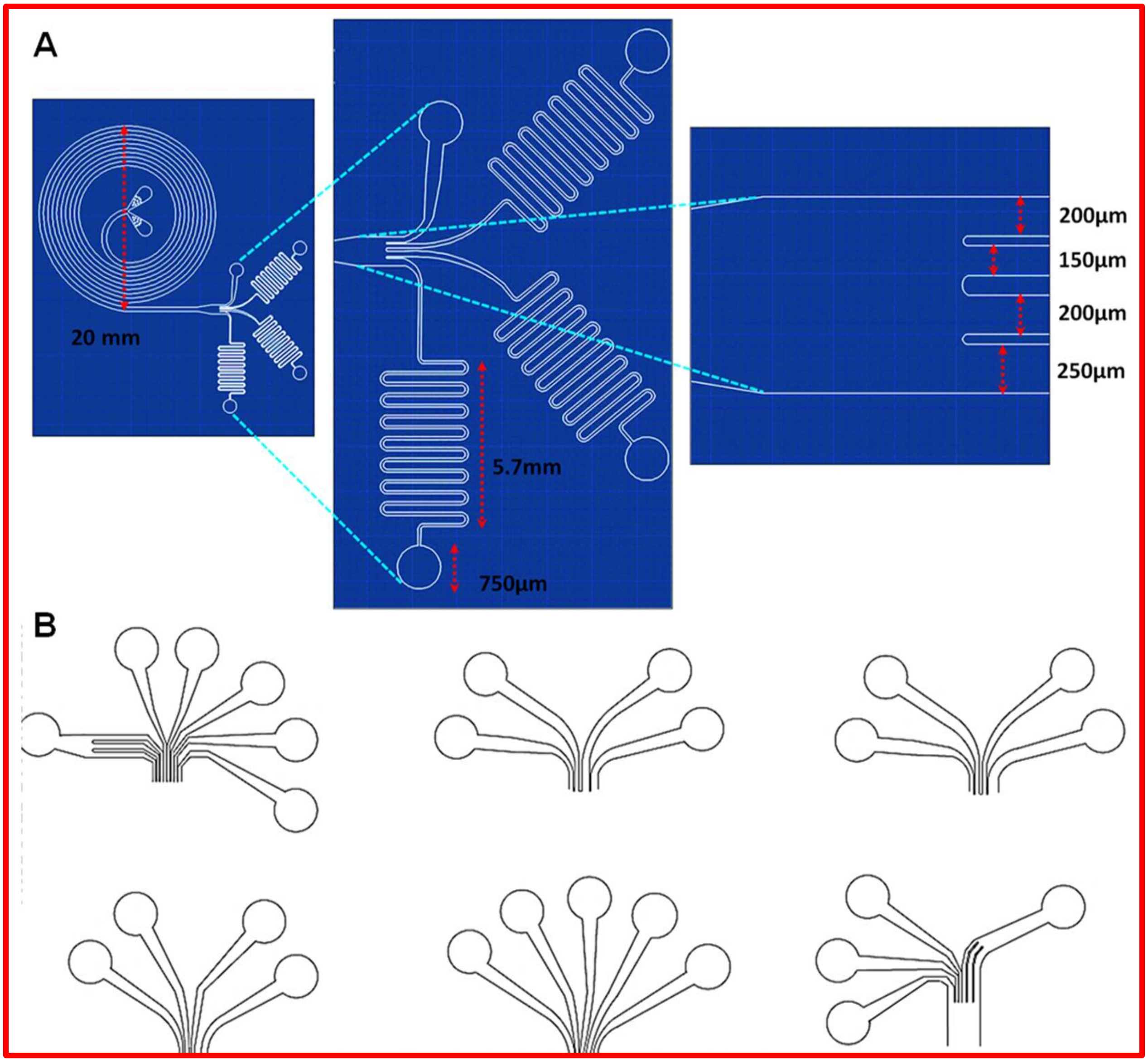

Figure S1. Inertial Sorter Optimization: parameters and devices studied during the 2nd generation device optimization to arrive at final spiral design (A) Optimized device dimensions showing resistance matching channels (B) Different outlet designs tested to facilitate optimal inertial cell sorting in stand-alone spiral.

3. Passive Mixing in Serpentine Channel

The serpentine configuration for the passive mixer has been chosen since the turns in the channels give rise to centrifugal Dean Forces that allow particles to cross fluid stream lines and become mixed. Dean forces increase with increasing fluid flow rate to lead to rapid mixing and

high mixing efficiencies.[27] Typically, for the microchannel-based passive mixer, flows through the device are primarily laminar with values of Reynolds' number, (Re), in the range of 0.01<Re<100 (Re~30 in the passive mixer studied)[13]. Because both viscous and inertial forces are appreciable in this range of Re, flow around the serpentine bend generates "secondary flows" in both the axial and radial directions thus increasing the interfacial area over which diffusion and mixing occur. The serpentine channel has a height of 100µm and a width of 250 µm and is capable of mixing particles at flow rates of 50 µL min-1 or higher. As the flow rate increased, mixing occurred sooner and was primarily dependent on chaotic advection rather than diffusion, which arises at slower flow rates where Dean forces are weak.

Preliminary evaluation of mixing was done by observing the mixing patterns of yellow- and blue- dyed buffer solutions generated in the mixing module. Using flow rates of 10-100 µL min-1, the mixing quality was characterized by the distance along the mixer required for the two distinct streams of yellow and blue, to become one well-mixed green stream (see SI Figure S1). The highly compact design of the mixer enabled fluids to be completely mixed by the third channel segment, at a length of ~4cm. Cancer cell labeling with EpCAM coated magnetic beads was examined through static experiments (PANC-1 cells and EpCAM-labeled beads in an Eppendorf tube) and on-chip experiments (on-chip passive mixing of PANC-1 cells and EpCAM-labeled beads subsequently flowed into reservoirs). Calculations demonstrate that for a cell to experience a magnetic force sufficient enough to pull it in the direction perpendicular to flow, and therefore allow it to be sorted, a cell of average diameter of 20 µm requires less than 1/3 bead coverage to be deflected to the desired magnetic sorter outlet (see SI Section 4).For each incubation time point (5, 10, 15 and 30 min), the quality of mixing was evaluated by immediately imaging the mixed effluent to quantify the extent to which cells were labeled with

magnetic beads. Cells were characterized as having no coverage, 1/3-2/3 coverage, and greater than 2/3 coverage (see SI Figure S3). In static conditions, with no other cells present, at least 15 minutes of incubation time was required to achieve at least 1/3 surface coverage on 85% of cells (SI Figure S6).

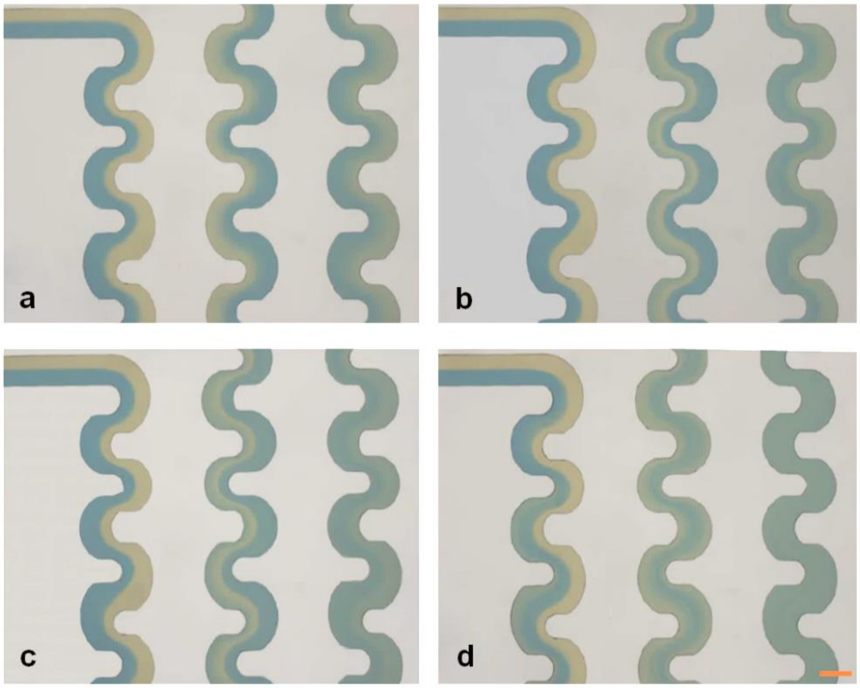

Figure S2. Mixing progression through passive mixer. Dyed PBS was used to demonstrate how increasing flow rates lead to mixing occurring closer to the beginning of the channel (a)10 µL min^{-1} (b)40 µL min^{-1} (c)70 µL min^{-1} (d)100 µL min^{-1}. Scale = 250µm.

4. Magnetic Bead Coverage Efficiency

Calculated Requirements. COMSOL modeling of magnets (K&J Magnetics, B224) shows a range of magnetic fields of 0.22-0.28T across the microchannel (Figure S2). These values are sufficient to saturate the magnetization of superparamagnetic microparticles as indicated by the hysteresis curve provided by the manufacturer. Therefore velocities are proportional to the

magnetic field gradient and magnetic particle movement can be measured and used for Stokes law calculations.

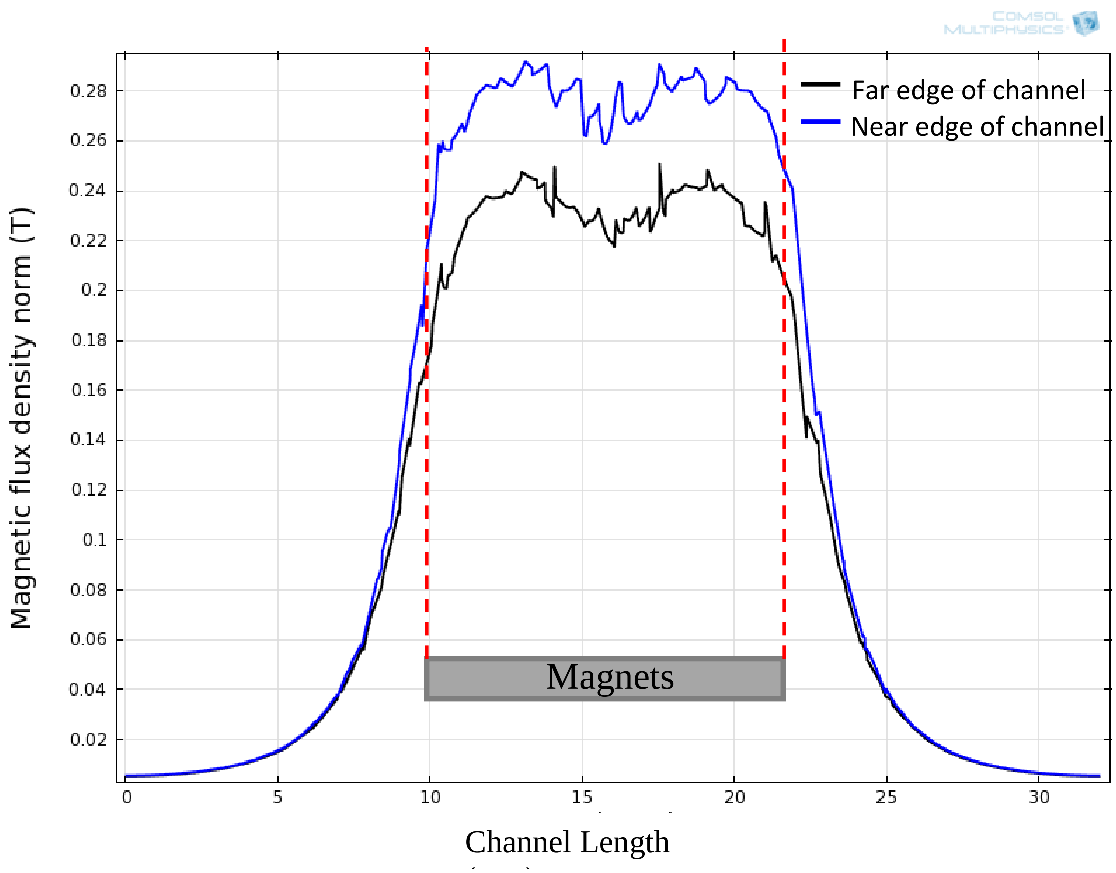

Figure S3. *Comsol* calculation of magnetic field strength at channel edges. Magnets (500mT) were positioned to be 1mm from the magnetic sorter channel.

The magnetic force on a magnetic particle in the laminar flow regime can be determined by applying Stokes law:

$$3\pi\eta u_t D_{bead} = F_D = F_{M(bead)} \quad \text{(S3)}$$

where η is the fluid viscosity, μ_t is the terminal velocity perpendicular to flow (which can be approximated by tracking bead movement across the channel), and D is particle diameter. Magnetic beads were placed introduced to a microfluidic channel and permanent magnets were placed 1mm from the channel edge. Particle movement was recorded using a high-speed camera.

Average velocities from tracked particles were determined to be 150µm s^{-1}, indicating a magnetic force of 1.4pN applied to each bead. To determine the cellular deflection as a result of attached magnetic beads, the magnetic force of the total number of beads will be equal in magnitude to the drag force of the cell. Again applying Stokes law where N represents the total number of attached beads,

$$NF_{M(bead)} = 3\pi\eta u_x D_{cell} \quad \text{(S4)}$$

This leads to a deflection velocity, u_x, of N · 7.5µm s^{-1}. Next cells were tracked while traveling in laminar flow through the microchannel and linear velocity was determined to be 125mm s^{-1} resulting in a residence time, or magnetic exposure time, of 0.2s (magnet length, 25mm). Then each magnetic bead is capable of deflecting the cell 1.5µm toward the magnet. Based on channel dimensions and streamlines, the maximum distance a cell needs to move is 100µm, therefore a minimum of 65 beads are required to ensure cell collection. Most observations of magnetic bead coverage of PANC-1 cells were minimum of 1/3 coverage which would be roughly 400 beads based on surface area. Thus, it is reasonable to assume that sufficient magnetic bead coverage is achieved for cell collection within the magnetic sorter module. The channel length is 25mm with a height of 100µm.

Minimum Magnetic Bead Coverage of Deflected Cells. Cells labeled with 1 µm magnetic and diluted in PBS were flowed through the magnetic sorter as previously described. The outputs of each outlet were examined via scanning microscopy to determine bead coverage required for collection. Figure S3 shows the minimum bead coverage necessary for cells to be deflected into the collection stream at a sample flow rate of 50µL min^{-1}. Video S1 provides further confirmation of labeled cells being pulled from the sample stream into the bottom outlet while remaining blood cells continue to upper waste outlet and are not deflected by the magnets.

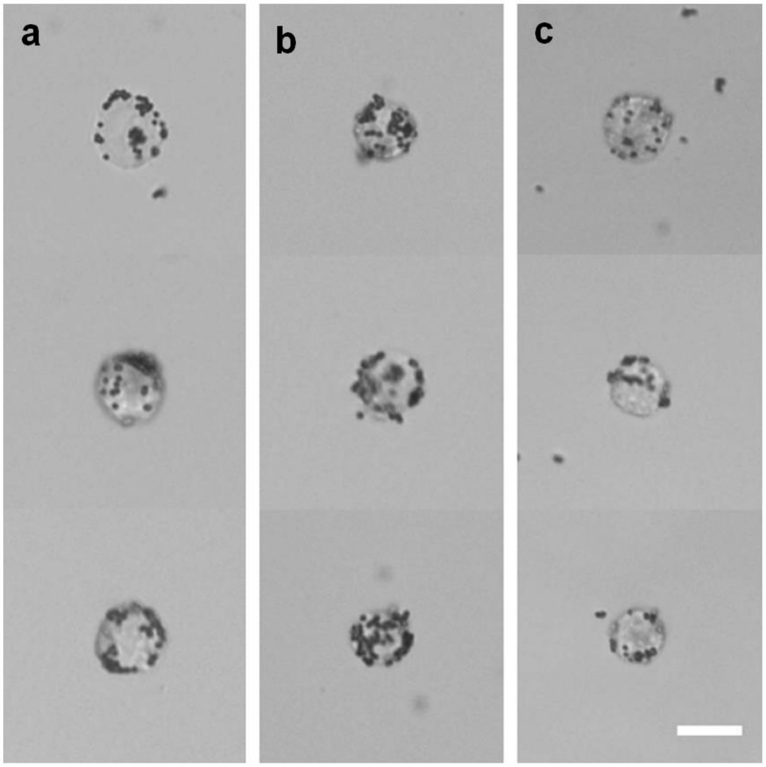

Figure S4. Minimum bead coverage required for magnetic collection. Cells were processed through magnetic sorter unit and each outlet was imaged. (a) Minimum coverage required for collection, (b) typical coverage in collection, (c) typical coverage in waste collection. Scale = 20 µm.

5. Experimental Labeling Efficiency and Magnetic Cell Sorting

To determine appropriate bead dilution and incubation time to ensure sufficient labeling of PANC-1 cells (1/3 surface coverage or higher of ~85% of cell), labeling experiments were carried out in buffer and blood. To test the on-chip mixing and labeling procedure, cells and diluted beads were flowed through the passive mixer at 100µL min^{-1} for a total volume of 1-1.5 mL. The cells and beads were allowed to incubate for the allotted time in the reservoirs with occasional rocking to prevent cell/bead settling and to enhance bead-cell mixing. As a negative

control, equivalent volumes of cell sample and beads were mixed in an Eppendorf tube. The tube was occasionally rocked to prevent cell/bead settling. After each incubation time, a drop of mixed sample was scanned on a slide at 20X and cells were counted based on their bead coverage (Figure S4). These experiments were performed with 1:10 and 1:5 bead dilutions and cell concentration of approximately 10^5 (Figure S5). Based on these results, 1:10 bead dilution was used and cell labeling was further tested in diluted blood to ensure bead-cell labeling efficiency was maintained even in blood. Figure S5 shows that 92% of cells have sufficient coverage after only 5 min incubation in reservoirs.

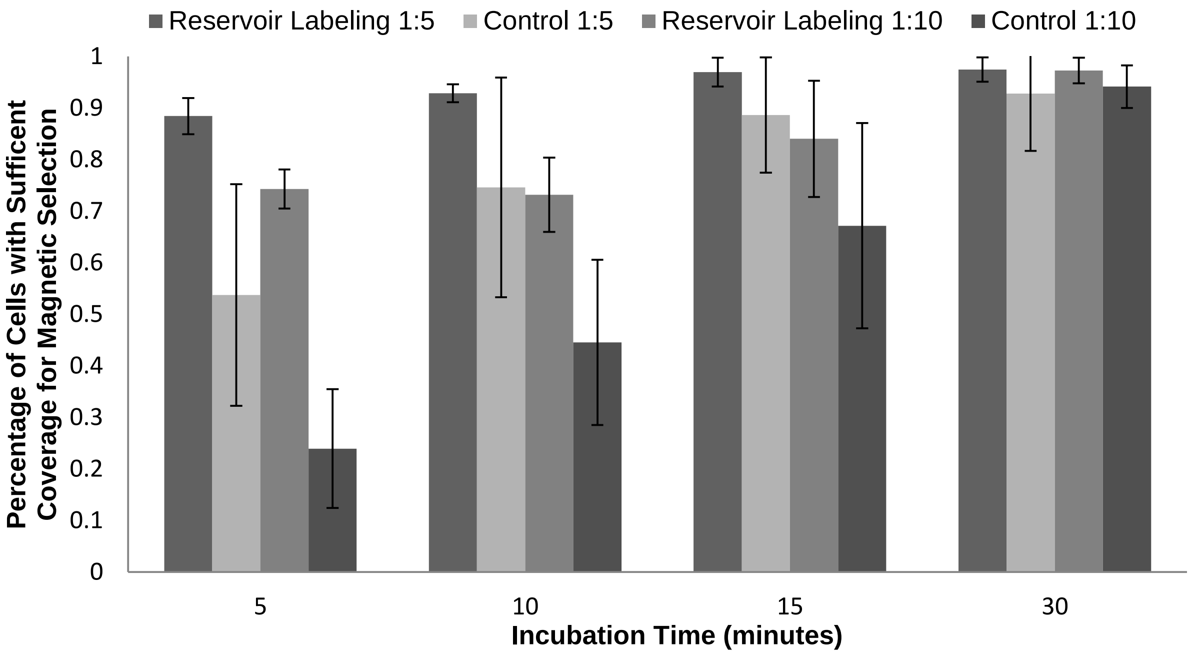

Figure S5. Magnetic bead labeling efficiency of PANC-1 cells. Bead concentration and incubation times were varied. Scanned images of cells were used to quantify bead coverage. Each experiment (data point) was repeated in triplicate. Bars indicate standard deviation.

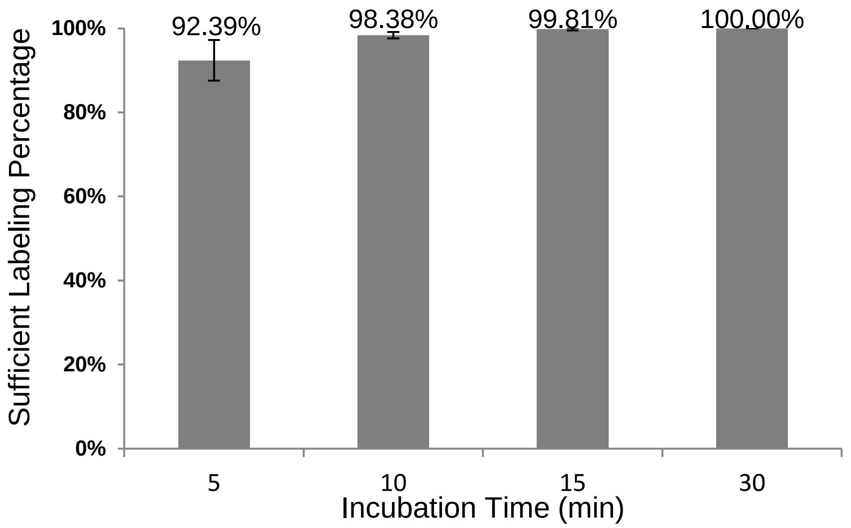

Figure S6. Incubation time required for bead-cell labeling. The percentage of PANC-1 cells (mixed in diluted blood) that were labeled within the reservoirs at times of 5, 10, 15, and 30 minutes. The beads were diluted 1:10 beads to buffer before entering the reservoir. (n=3, bars indicate standard deviation)

To determine the flow conditions that would facilitate the highest purity rates for cancer cell sorting using the magnetic sorter module, two conditions, one at 50µl/min bottom-17 µL min^{-1} top (50 µL min^{-1} bias), and the other at 75 µL min^{-1} bottom-25 µL min^{-1} top (75 µL min^{-1} bias) were tested. For the 50 µL min^{-1} bias condition, contamination was 2.35% ± 2.72 and efficiency was 88.39% ± 5.79 in the collection channel. For the 75 µL min^{-1} bias setup, WBC contamination decreased to less than 0.5% but the recovery efficiency was also reduced (figure S6). Since the purity was higher using the 75 µl/min bias conditions this setup was finally implemented.

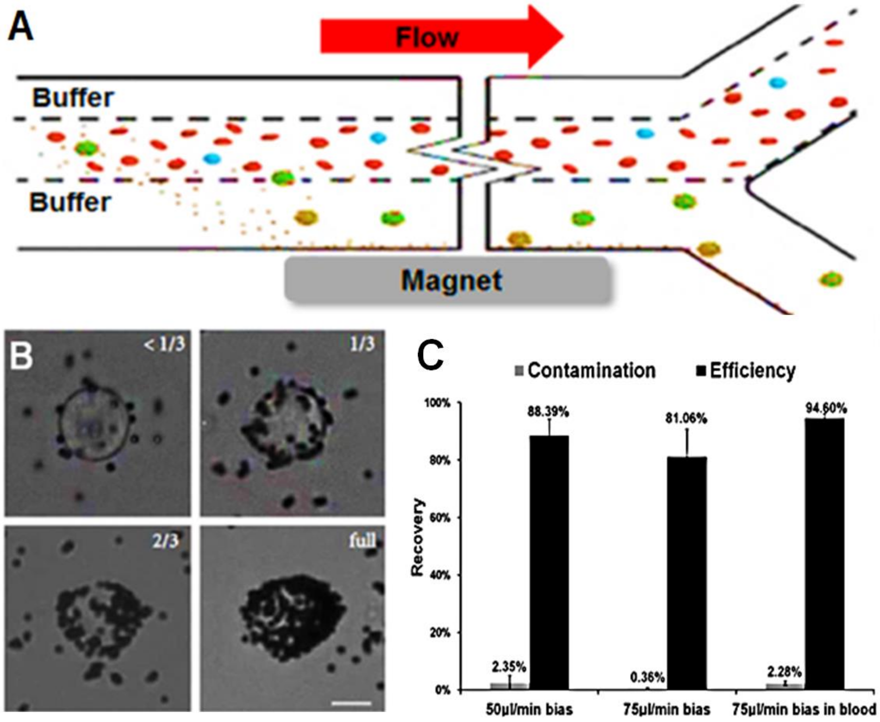

Figure S7. Magnetic Sorter Operation (A) Schematic of device operation. Only magnetically labeled cancer cells get attracted and flow to the collection outlet. (B) Representative images of the categories of bead coverage used to characterize labeling of cells collected (C) Graph showing the recovery and contamination rates of PANC-1 cells and WBCs spiked in buffer for 50 and 75µL min^{-1} bias conditions and diluted blood for 75µL min^{-1} bias condition.

6. *Bead/Antibody Reactivity*

Assuming that magnetic sorting of CTCs may be useful for commercial applications of point-of-care testing the stability of the 1µm streptavidin coated superparamagnetic beads were evaluated up to 100 days after the beads were functionalized with biotinylated EpCAM per manufacturer's instructions. Day 0 indicates a sample taken after 30min of incubation with the newly functionalized beads. This experiment was repeated incrementally using the same stock of beads that had been functionalized on Day 0 and kept at 4°C while not in use. For each time point, 100µl of PANC-1 cells (~10^6/ml) were incubated for 30min at room temperature with 10µl of functionalized beads (original stock concentration). As seen in Figure S7, beads remain active

through Day 100 and continue to show the same full surface coverage of PANC-1 cells. This indicates the functionalized beads remain active and capable of cell antigen interaction when stored at 4°C.

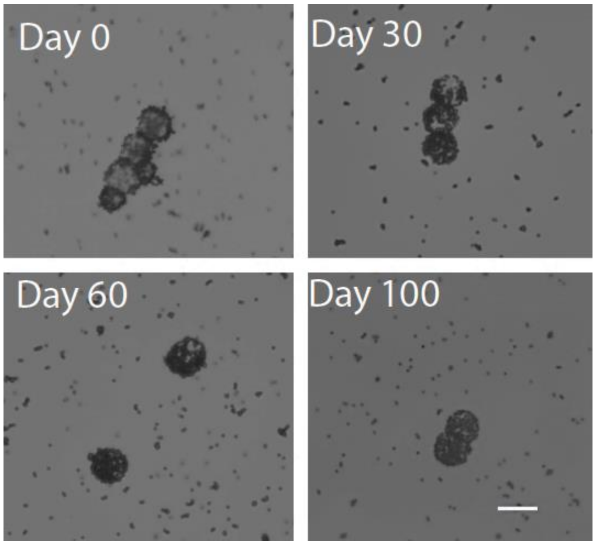

Figure S8. Examination of the prolonged reactivity of antibody coated beads. PANC-1 cells were treated with anti-EpCAM coated beads for 30min and then imaged. Time points indicate the numbers of days after beads were initially conjugated to EpCAM antibody. Beads were stored at 4°C and original stock concentration for the duration. Scale bar = 25 µm

7. Cell viability

To ensure cells processed through the integrated magnetic device would be viable for further cellular studies, the viability of cells was assessed through the MTT assay (Figure S7). PANC-1 cells were either seeded directly into a 96-well plate (Control), processed through the integrated device in media without magnetic beads (Test 1), or processed through the integrated device in media with the addition of magnetic beads (Test 2). Magnetic beads were exposed to UV light for 8 hours prior to use in these experiments to promote experimental sterility. After collecting the output of the integrated device, the processed cells were also plated in 96 well plates at 4000 cells/well. Every 24 hours, cells were subjected to MTT protocol and absorbance at 570nm was

recorded. Control Day 1 was used as the basis to normalize the results. Media was changed on Day 3. This experiment was repeated 3 times and showed similar growth patterns. Cells that were processed through the device were viable and resulted in nearly the same growth rate as the control cells.

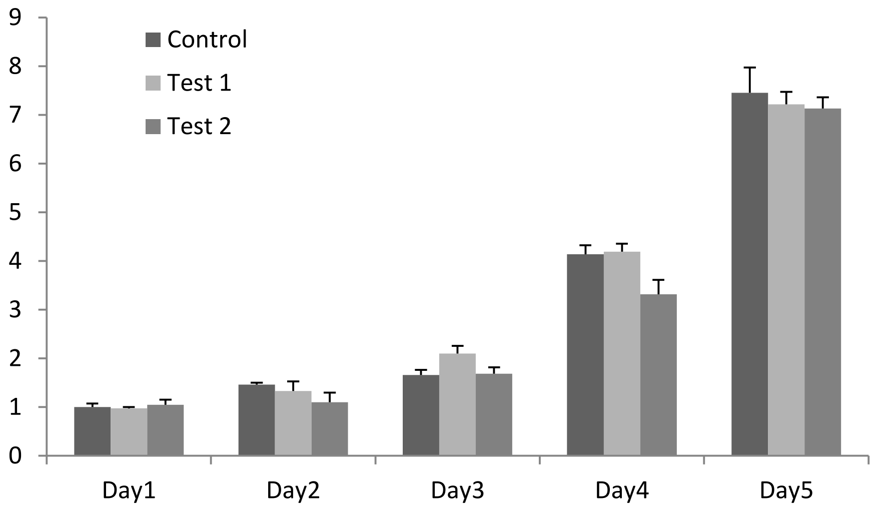

Figure S9. MTT assay to determine cell viability. Cells were processed through the integrated device either without magnetic beads (Test 1) or with magnetic beads (Test 2). Control cells were unprocessed. Then cells were plated in 96 well plates and grown for 5 days – with MTT assay performed on one plate per day to assess growth. Growth was normalized to control cells on Day 1. (n=5, bars indicate standard deviation)

Table S1. Data for individual patient samples showing patient statuses. CTC counts from the integrated microfluidic chip are also reported.

DATE	PATIENT ID	VOL. (mL)	CTCs/ mL	WBCs/ mL	AGE	SEX	DISEASE STAGE
3/3/2014	P1	1.2	938	7	73	F	Metastatic to lymph nodes outside of surgical field
4/14/2014	P2	1.0	80	0	81	F	Locally Advanced/unresectable
4/21/2014	P3	1.0	16	0	75	M	Likely PDA/resectable (pending Pancreatitis)

**4/21/2014**	P4	1.0	22	0	50	F	Locally PDA
**7/29/2014**	P5	1.3	141	12	63	M	pT4 N1 MX-Stage III
**7/29/2014**	P6	1.4	190	25	53	M	PDA Stage IV
**8/4/2014**	P7	1.2	38	0	58	F	PDAM Stage IB-IV
**8/4/2014**	P8	1.2	80	35	60	M	PDA Stage IV
**8/11/2014**	P9	0.7	280	49	66	M	Metastatic Panc Adeno to liver Stage IV
**8/11/2014**	P10	0.6	58	389	63	M	Locally advanced/borderline resectable
**8/12/2014**	P11	1.4	20	14	40	M	Resected pT3N1- Stage IIB
**8/18/2014**	P12	1.2	48	12	81	M	Possible PDA, renal mass
**8/18/2014**	P13	1.0	14	0	61	M	Metastatic Panc Adeno Stage IV
**8/18/2014**	P14	1.4	113	51	65	M	Metastatic Panc Adeno Stage IV

Table S4. Data for individual patient samples either miRNA or mRNA profiled. CTC counts from the integrated microfluidic chip are also reported.

**micro RNA Profiling**					
**Date**	**ID**	**Vol. (mL)**	**CTCs/mL**	**WBCs/mL**	**Stage**
02/09/15	P1	5	7	2	Borderline resectable-Resected
02/09/15	P2	5	15	1	Borderline resectable

05/19/15	HC1	5.5	0	0	N/A- Healthy Control
**mRNA Profiling**					
**Date**	**ID**	**Vol. (mL)**	**CTCs/mL**	**WBCs/mL**	**Stage**
1/12/15	M1	6.5	11	0	Metastatic PDAC-deceased Aug 2015
1/26/15	L1	6.5	1	3	Locally advanced PDAC
12/15/14	B1	12	9	0	Borderline resectable
1/12/15	B2	5	161	27	Borderline resectable-Resected
12/15/14	B3	12	6	0	Borderline – Eventually developed metastasis
12/15/14	M2	12	9	0	Metastatic PDAC- Showed Progression
01/20/15	L2	5.5	12	0	Locally advanced PDAC concerning for metastasis
12/8/14	M3	7	62	1061	Metastatic- Resected – deceased Mar 2015

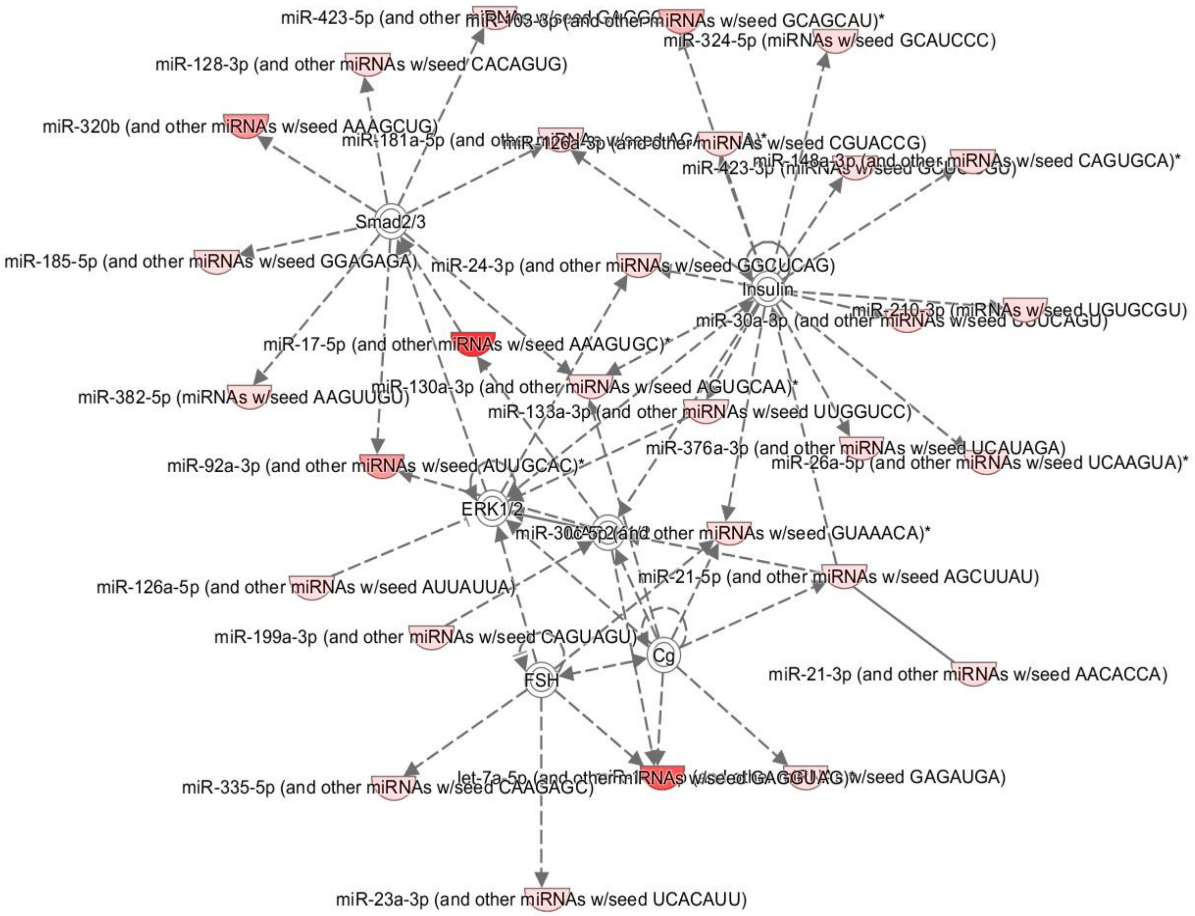

Figure S10. Network generated in IPA from CTC MiRNA profiling infers relationships among miRNAs with highest levels of expression. Lines, referred to as “edges” indicate inferred relationships between molecules. MAP2K1/2 and Smad2/3 pathways appear to be impacted based on the miRNA expression profile of the PDAC CTCs.
